# Have We Any Cholagogues?—Alumni Prize Essay

**Published:** 1874-04-01

**Authors:** George N. Fuller

**Affiliations:** Webster City, Iowa


### MEDICAL EXAMINER.

A

*Semi-Monthly Journal of Medical Sciences.*

EDITED BY N. S. DAVIS, M.D., AND F. H. DAVIS, M.D.

No. VII. CHICAGO, APRIL 1, 1874. VOL. XV.

### Original Communications.

### HAVE WE ANY CHOLAGOGUES?—ALUMNI PRIZE ESSAY.

BY GEORGE N. FULLER, M.D., WEBSTER CITY, IOWA.

THIS has lately been strongly disputed; yet many, from their observation of the effects of certain medicines, still think that there is some foundation for the ancient belief in them.

We find some confusion, however, in the signification attached to the word. It is usually defined "medicines increasing the flow of bile." It is often used as if signifying "medicines increasing the *secretion* of bile."

Mercury is usually placed at the head of this class of medicines, when such a class is recognized; yet there is much dispute in regard to the effect of that drug. Stille quotes, and apparently indorses, the language of Thudicum, that "calomel is not a cholagogue, but decreases the secretion of bile."* Yet he elsewhere says: † "The repeated use of this medicine (calomel) is known to produce derangement of the hepatic function;" implying that it is by overstimulation of that function. He also quotes this language of Budd: ‡ "When the liver has become accustomed to the stimulus of mercury, no other medicine will sufficiently excite its action. * * It increases the activity of the liver at first, but seems to leave it weaker than before," etc.

It is impossible to explain the contradictory language of Stille, except by supposing that he intended to give

* Page 683, Vol. II., Third Edition *Ther. and Mat. Med.*

† Page 710. ‡ Pages 711–712.

a summary of the diverse opinions held upon this subject.

This diversity is certainly great. Some think that the flow of bile is much increased by mercury and other agents; and others that it is unaffected or decreased by their use.

Perhaps the most famous experiments of the many made on this subject, were by a committee of Edinburgh physicians, upon dogs, with different substances, including calomel, taraxacum, and podophyllin. Their investigations went far to overturn the belief in the specific action of these substances on the secretion of bile.

There are, however, some objections to these experiments. They were made upon lower animals; and, however judiciously selected these animals may be, we cannot be certain that the effect upon them is the same as upon man. The experiments were made by the establishment of biliary fistulæ. No doubt this was carefully done; yet the effect of the drugs may not have been the same as if the bile-ducts had been left undisturbed in their connections with the duodenum. They were made in a state of health, so far as known, of the animals experimented upon; the action may be thought different from what it would be in pathological conditions. The last I do not consider a serious objection. The physiological action of a drug is usually a fair indication of the character, at least, of its effect in pathological conditions. In the latter case, the action may sometimes appear greater; but I am not aware that any drug has a stimulating action upon an organ or function in a diseased condition, on which it has no effect in health.

I have performed some experiments to obtain information on this subject, to which the first two objections do not apply. How far they will aid in the solution of this problem, is for my co-laborers in this field to judge.

The experiments were made by testing liquid drained from fæces for bile, by Pettenkofer's test, substantially as described in *Dalton's Physiology.** I made a solution of cane sugar, one part; water, four parts. One drop of this solution was added, in a test-tube, to about a fluid drachm of the liquid to be tested, and an equal quantity of sulphuric acid added. If bile was present, a bright, cherry-red color was at once produced, soon followed by a lake, and that by a rich purple.

I first practiced testing water with which I had mingled ox bile. I found the tests quite plain and reliable, and not affected by slight variations in the proportions of the reagents. If I added the sugar solution without adding the bile, the sulphuric acid produced a brownish red color, which gradually became a distinct brown. If too much sugar was added, this would obscure the reactions when bile was present; but with care, two drops of bile to a fluid drachm of water would produce the reactions promptly. One-half, or even one-fourth, of this proportion of bile would produce them, but with some delay.

I practiced these tests with ox bile until I considered myself familiar with the different reactions. I also repeated them at different times during the course of my experiments, to

* Page 185, Third Edition.

refresh my recollection, to test reagents, and to observe how the different liquids were affected by temperature.

Dalton states that other substances present, acted upon by the sulphuric acid, may obscure the reactions; but that * "no other substance is liable to be met with in the intestinal fluids or blood, which would simulate the reactions of the biliary matters." He also says that † the red color alone is not sufficient as an indication of bile. It is the lake and purple colors alone which can be regarded as really characteristic of the biliary reactions."

In testing liquid filtered from fæces, I found that the action of the acid produced different shades of brown, more or less resembling lake and purple. These I concluded to be from the effect upon bile in greater or less degrees of decomposition; it might have been partly from matters derived from other sources. These reactions often resemble the brown produced by the action of the acid upon the sugar solution; and great care is sometimes required to distinguish them. I do not think that I committed any errors in this way, however. In most of the experiments it will be noticed that diluted solutions were tested, and gave reactions weaker in proportion to the degree of dilution. In such a case, the reactions could not have been from the action of the acid upon sugar, as the amount of the sugar solution added in each case was as nearly equal as it could be made.

I took this method of testing fæces, because I was at a distance from the means of making more delicate tests, or quantitative analyses. With the means at my command, the tests could not be made sufficiently accurate to indicate the exact proportion of bile discharged. They are useful, principally, for the comparison of the discharges following the use of certain substances with those occurring naturally.

It was only after some experience that I arranged the proper proportion of water to be added, and other minutiæ. Had there been time, I should have completed the series in this uniform manner. I was unable to do this; but these variations do not affect the general result. In making the experiments, the fæces were received into ordinary self-sealing fruit-jars, weighed, and mixed with water. They could thus be treated without being very offensive. After they were mixed with water there was less odor, and the filtered liquid was but little offensive.

The experiments are numbered merely for reference here, without regard to the time when they were made; but I was careful to make no experiment in less than six days after a previous one with drugs; also, to make none when my system did not appear to be in a healthy condition.

EXPERIMENT WITH NATURAL FÆCES — *First Experiment, May 20th*, 1873.—Weight of fæces four ounces; to three and a half ounces added seven fluid ounces of water; mixed, and filtered. Reactions not biliary; reddish yellow, followed by reddish brown.

*Second Experiment, July 15th*, 1873. —Weather very warm; weight of fæces nine and a half ounces; added same number of fluid ounces of water, and mixed; stood over night and fer-

* *Phys.*, Third Edition, p. 187.

† *Phys.*, Third Edition, p. 186.

mented; liquid more than usually separated from solid matters. Biliary reactions quite distinct and rapid; colors rather dark. By accident, the dilutions not tested.

*Third Experiment*, *July* 25*th*, 1873.—Bowels rather constipated for three days; weight of fæces two ounces; added six fluid ounces of water; mixed, and filtered. Biliary reactions pretty prompt; colors rather dark; lake and purple not very distinct, but perceptible. Diluted one-half, the cherry red was tolerably distinct; the others not.

*Fourth Experiment*, *July* 26*th*, 1873.—Free evacuation; weight of fæces eight ounces; added twice the number of fluid ounces of water; mixed, and filtered. Obtained rather dark cherry-red and brown, resembling lake and purple. Diluted one-half, reactions nearly as distinct; again diluted one-half, a brownish red tinge only.

*Fifth Experiment*, *August* 5*th*, 1873.—Bowels rather constipated for several days; last operation thirty hours before; fæces nearly solid; weight five ounces; treated same as last. Obtained pretty prompt and distinct cherry-red, and faint but perceptible lake and purple. Diluted, the reactions were perceptible, but not distinct.

*Sixth Experiment*, *August* 7*th*, 1873.—Bowels natural; fæces of medium consistence; weight eight ounces; treated same as last. No biliary reactions.

[Other experiments with natural fæces will be found given as parts of some of those with drugs.]

EXPERIMENTS WITH SALINES—*Seventh Experiment*, *December* 4*th*, 1872.—Usual evacuation at 9 A.M.; two hours after, took mag. sulph. and pot. bitart., of each one half ounce; water, four ounces: between 1 and 2 P.M., had three copious evacuations of liquid fæces; weight twenty ounces; added ten fluid ounces of water, and mixed. No distinct biliary reactions.

*Eighth Experiment*, *January* 29*th*, 1873.—Usual evacuation at 8 A.M.; between 6:30 and 11:30 P.M., took mag. sulph. and pot. bitart., of each six drachms, in divided doses: considerable peristaltic motion, and some pain, from 8 P.M. to 12:30 A.M.; between the last time mentioned and 1:30 A.M., there were three rather copious discharges, mostly liquid; weight sixteen ounces; added same number of fluid ounces of water; mixed, and filtered. Obtained cherry-red promptly; lake and purple rather slow, and not distinct. Diluted, gave reactions nearly as distinct; again diluted, perceptible, but much less distinct.

*Ninth Experiment*, *May* 29*th*, 1873.—Usual evacuation at 9 A.M.; at 5 P.M., took mag. sulph. and pot. bitart., of each two drachms, in solution; and at 8:30 a similar dose: between 6 and 10 P.M., some nausea, peristaltic motion, and pain, perceived; slept naturally till 5:30 A.M.; at 6 and 9 A.M., had somewhat copious semi-liquid discharges; color dark greenish brown; weight about sixteen ounces; to twelve ounces added same number of fluid ounces of water; mixed, and filtered. Obtained faint cherry-red, indistinct lake, and no purple. Diluted one-half, gave reddish brown only.

*Tenth Experiment*, *August* 14*th*, 1873.—A.M., natural evacuation of buttery fæces; weight six ounces; added twice the number of fluid ounces of water; mixed, and filtered. Obtained only brownish red color, growing darker. At 6 A.M. next day, took mag. sulph. and pot. bitart., of each four and a half drachms, in solution: between 8 and 9 A.M., some peristaltic motion perceived; at 10 A.M., took similar dose: between 11 and 12, had three copious evacuations; fæces mostly liquid; weight twenty-four ounces. Treated as last; obtained only slight tinge of brown.

EXPERIMENT WITH ALOES—*Eleventh Experiment*, *January 13th*, 1874.—Usual evacuation, A.M.; at 6:30 P.M., took aloe soc., four grains, and ext. hyosc., one grain; at 9:30 P.M., took similar dose: perceived slight pain in bowels, and peristaltic motion, between 4 and 6:30 A.M.; between 6:30 and 9:30 A.M., three copious evacuations of buttery fæces; color light reddish brown; weight twenty-five ounces or more; second evacuation, nine ounces; to this added twice the number of fluid ounces of water; mixed; kept in warm room forty-eight hours, and filtered. Reactions variable, but not resembling those of bile.

EXPERIMENTS WITH FRUIT—*Twelfth Experiment*, *February 8th*, 1873.—At 4 P.M., ate two good-sized sour apples; at 8 P.M., the same: between 6 and 7, also between 8:30 and 10 P.M., there was some sensation of fullness, and peristaltic motion of the bowels. At 8 A.M., passed ten ounces of dark brown fæces; rather more, and rather less solid than usual; added same number of fluid ounces of water; mixed, and filtered. Obtained prompt and distinct biliary reactions. Diluted one-half, equally so; again diluted, not as prompt, but retty distinct.

*Thirteenth Experiment*, *January 20th*, 1874.—A.M., natural evacuation of light brown, somewhat buttery fæces; weight eleven ounces; added eighteen fluid ounces of water; mixed; kept in warm room forty-eight hours, and filtered. Obtained very slight tinge of cherry-red, followed by reddish brown. At 3 P.M., ate three medium-sized, moderately sour apples; at 9 P.M., did the same: at 8 A.M., passed fæces; weight nine ounces; rather darker, and less solid than the day before; added eighteen fluid ounces of water; mixed; kept in warm room forty-eight hours, and filtered. Reactions resembled the biliary; not distinct.

EXPERIMENTS WITH TARAXACUM—*Fourteenth Experiment*, *December 29th*, 1873.—At 9 A.M., natural evacuation of semi-solid brown fæces; weight, five ounces; added twice the number of fluid ounces of water; mixed; kept in warm room ten hours, and filtered. Obtained rather dark cherry-red, and shades of brown resembling lake and purple. During the afternoon, took fluid ext. taraxacum, three drachms, in two doses: at 9 A.M. following, passed three ounces of brown, nearly solid, fæces. Treated same as last; tests made in too small tube, and not reliable, but seemed to resemble biliary rather more than the day before. At 9 A.M. following, passed four and a half ounces of rather light brown, nearly solid, fæces; treated same as last, only kept twenty-four hours in warm room. Very carefully tested, the colors resembled biliary but little.

*Fifteenth Experiment*, *February 2d*, 1874.—At 9 A.M., natural evacuation of light brown, nearly solid, fæces; weight six ounces; treated as last, only kept forty-eight hours in warm room. Obtained dark, indistinct cherry-red, and shades of brown resembling lake and purple. Diluted, gave reactions nearly as distinct; again diluted, perceptible, but not as distinct. During that and the next day, took fluid ext. tarax., six drachms, in divided doses: at 10 A.M. following (4th inst.), passed three ounces fæces; rather less, lighter color, and more solid, than usual. Treated as the last: obtained similar reactions; perhaps a little more distinct.

*Sixteenth Experiment*, *February 9th*, 1874.—At 9 A.M., usual evacuation of light brown, nearly solid, fæces; weight six ounces. Treated as last, except stood but twenty-four hours before filtering: reactions prompt, and resembling biliary. Diluted one-half, less prompt and distinct, but perceptible. During that and following day, took fluid ext. tarax., one ounce, in divided doses: at 8 A.M. next day (11th inst.), passed seven ounces of light brown fæces, nearly solid. Treated same as last: reactions similar, but rather more distinct.

EXPERIMENTS WITH CALOMEL — *Seventeenth Experiment*, *January 15th*, 1873 —Usual evacuation at 9 A.M.; at 3 P.M., took hydrarg. chlor. mit., twelve grains: between 5 and 6 P.M., perceived some nausea, and peristaltic motion. At 6:30, took mag. sulph. and pot. bitart., of each one drachm, in solution: between 7 and 8 P.M., had one solid and three copious liquid discharges; weight twenty ounces; added same number of fluid ounces of water, and mixed. Obtained cherry-red promptly; and lake and purple with a little delay; all quite distinct. Diluted one-half, the reactions were nearly as prompt and distinct; again diluted, they were perceptible, but quite indistinct.

*Eighteenth Experiment*, *December 19th*, 1873.—At 9 A.M., rather free natural evacuation of dark brown, buttery fæces; weight eight ounces; added twice the number of fluid ounces of water; mixed, and filtered; room cold; liquid probably chilled, not frozen; filtered liquid not as dark as usual. Obtained only brownish red colors. At 10 P.M., took hydrarg. chlor. mit., twelve grains: between 3:30 and 5 A.M., perceived some nausea, peristaltic motion, and pain; between 5 and 6:30 A.M, five tolerably copious evacuations of thin brown fæces; weight thirty-two ounces or more; nine ounces, including part of first three and all of fourth evacuation, were treated same as last; temperature about the same. Reactions similar; hardly as distinct.

This experiment was not very satisfactory, but showed that no very marked increase of biliary flow had taken place, although the appearance of the fæces was what is considered characteristic of "bilious passages." To ascertain whether cold had prevented the reactions, I tested a mixture of ox bile and water, which had been frozen. I found that the reactions were produced, but that the colors were not exactly the same. I also tested a mixture of ox bile and water, which had been exposed to sufficient cold to chill it—in fact, to freeze a small portion of it. I found that this did not appreciably change the reactions. As this was fully as great a degree of cold as that to which the specimens were exposed, I concluded that this did not modify the results, except so far as it hindered the complete solution of the secretions in the water added.

*Nineteenth Experiment, January 26th*, 1874.—At 8 A.M., natural evacuation of semi-solid, light brown fæces; weight six ounces. Treated as last, only kept in warm room forty-eight hours before filtering: obtained slight cherry-red color, and shades of brown considerably resembling lake and purple. Diluted one-half, colors nearly as distinct; again diluted, much less distinct. At 10 P.M., took hydrarg. chlor. mit., twelve grains: between 5 and 7 A.M., had four tolerably free evacuations of brown fæces, mostly liquid; weight twenty-five ounces or more. Portions of second and third evacuations, weighing eight and a half ounces, were treated as last: obtained similar reactions from all the dilutions; perhaps a little more distinct.

There was considerable difference in the reactions, at different times, in all the classes of experiments. Probably this difference was due, to some extent, to variations in temperature and in modes of manipulation; yet the variations were not greater than every one has observed in the amount, color, consistence, and odor, of healthy fæces.

The amount of bile discharged was not increased by taraxacum. This agent produced a constipating, rather than a laxative, effect; and to this I attribute the slight increase, in proportion, of bile by its use. Had there been an actual increase of the bile discharged, the proportionate increase, with less fæces, would have been much greater.

The proportion of bile discharged was not increased by calomel or other cathartics; was sometimes probably decreased by them, especially by aloes and the salines. In regard to aloes, this is only inferred; in regard to the salines, this is shown by the tenth experiment, and may be inferred from the seventh and ninth. After the use of the latter, bile was detected only after prolonged peristaltic action, and then in only small amount. Peristaltic motion alone will not produce free discharge of the bile; if it would, there would have been such in the eighth experiment, according to my painful recollection.

When, after the use of cathartics, the proportion of bile detected was the same, of course the amount was greater, as the amount of fæces was greater. This was, probably, because much of the bile in the intestines was expelled, and not from increased biliary secretion. Had it been the latter, the proportion of bile would have been greater. The largest amount of bile detected was not more than an ounce and a half. Making all due allowance for errors, and for the obscuring of reactions by other substances, this is less bile than is contained, at any time, in the intestines.

The secretion of bile was probably not increased by any of the agents used. It may be objected, that more bile was secreted, and discharged into the intestines, and then absorbed, as most of the bile usually is. But bile is usually changed or decomposed by reactions with the other contents of the intestines before absorption. By the action of a cathartic, the contents of the intestines are hurried along too rapidly for either change or absorption of an increased amount of bile. In the natural condition, nearly all the liquid contents of the intestines are absorbed. When a cathartic is given, the discharges are liquid, partly because more liquid is secreted or exuded, and partly because increased peristaltic motion does not give as much time for the liquids to be absorbed. Physiologists estimate the normal amount of bile secreted at about two and a half pounds in twenty-four hours, or about a pound in ten hours. If the flow of bile is increased by the use of calomel, it seems strange that all this bile should be absorbed, while the other liquids are discharged in such quantities.

It may also be said that there was more bile discharged into the intestines, but that it had not passed far enough down to affect the fæces. It may be difficult to prove that this was not the case; but we have no reason to believe that it was.

The liquid condition and brown color of the discharges produced by calomel are not produced by bile. If they were, the proportion of bile would be quite large; but there was very little detected, although the discharges were of the traditional color and consistence.

These results are different from what I expected, and from what I announced from a much shorter series of experiments; but they have been carefully reached, and agree with those others have obtained in different ways. Clinically, especially in a miasmatic region, mercury may often be very useful, and may sometimes produce good effects which other remedies will not; but, in accounting for this effect, perhaps false pathology and false therapeutics have been mingled.

Much is laid to "torpid liver," which is quite as much inaction of all the nutritive functions, especially those of the intestinal glands. An impression upon these, often best made by calomel, may bring about a more natural condition, and the biliary secretion not be affected, directly, at all.

When there is really suspended secretion of bile, from hepatic congestion, cathartics may indirectly give relief by reducing portal congestion; and calomel will often do this more gently and thoroughly than any other cathartic.

But this series of experiments tends to confirm the conclusions of others, that we have no medicines possessing a specific power to stimulate the secretion or flow of bile.

February 12, 1874.

---

EFFECTS OF THE LONDON FOG.—The unusual density and duration of the recent fogs in London were exceedingly disastrous, causing the death of many persons affected with cardiac and respiratory diseases, and greatly augmenting the death-rate. There were, altogether, about fifty patients taken to the various hospitals on account of accidents due to the fog. The number of deaths from diseases of the heart and lungs was 764 the week of the fog, and only 560 the previous week.—*N. Y. Med. Jour.*

